# Flavonoids intake and weight-adjusted waist index: insights from a cross-sectional study of NHANES

**DOI:** 10.3389/fnut.2024.1400726

**Published:** 2024-06-18

**Authors:** Shuang Zu, Meiling Yang, Xiude Li, Hanhan Wu, Xunliang Li, Yunshan Fan, Deguang Wang, Bao Zhang

**Affiliations:** 1 Department of Nephrology, The Second Affiliated Hospital of Anhui Medical University, Hefei, Anhui, China; 2 Department of Clinical Nutrition, The First Affiliated Hospital of Anhui Medical University, Hefei, Anhui, China; 3 Department of Gastroenterology, The First Affiliated Hospital of Anhui Medical University, Hefei, Anhui, China

**Keywords:** flavonoids intake, weight-adjusted waist index, NHANES, abdominal obesity, flavonoids dietary recommendation

## Abstract

This study conducted data on 15,446 adults to explore the impact of flavonoids on weight-adjusted waist index (WWI). This was a nationwide cross-sectional study among US adults aged 20 years or older. Dietary intake of flavonoids was assessed through 24-h recall questionnaire. WWI was calculated by dividing waist circumference (WC) by the square root of weight. We utilized weighted generalized linear regression to evaluate the association between flavonoids intake and WWI, and restricted cubic splines (RCS) to explore potential non-linear relationships. Our findings indicated that individuals with lower WWI experienced a notable increase in their consumption of total flavonoids, flavanones, flavones, flavan-3-ols, and anthocyanidins intake (*β* (95% CI); −0.05(−0.09, −0.01); −0.07(−0.13, 0.00); −0.07(−0.11, −0.02); −0.06(−0.11, 0.00); −0.13(−0.18, −0.08), respectively), with the exception of flavonols and isoflavones. Additionally, consumption of total flavonoids, flavonols, flavanones, isoflavones, and flavan-3-ols had a non-linear relationship with WWI (all *P* for non-linearity < 0.05). Furthermore, the effect of total flavonoids on WWI varied in race (*P* for interaction = 0.011), gender (*P* for interaction = 0.038), and poverty status (*P* for interaction = 0.002). These findings suggested that increase the intake of flavonoids might prevent abdominal obesity, but further prospective studies are requested before dietary recommendation.

## 1 Introduction

Obesity arises from the disproportionate accumulation of body fat, a consequence of energy intake surpassing energy expenditure. The prevalence of obesity has reached epidemic levels globally, impacting a growing population of people (1, 2). Abdominal obesity, a form of obesity characterized by an accumulation of fat specifically in the abdominal region, is associated with an increased risk of metabolic imbalances, diabetes, high blood pressure, and cardiovascular diseases, beyond overall amount of body fat (3). These elements are poised to create a public health concern, necessitating intervention strategies once the contributing factors have been assessed. Research indicated that people with a high volume of abdominal fat and a large waist circumference (WC) not only have more intra-abdominal fat but also larger subcutaneous fat cells, accompanied by adipose tissue that is dysfunctional and inflamed (4). Numerous indicators have been utilized to assess obesity, with a particular focus on the detrimental intra-abdominal fat mass. While Body Mass Index (BMI) remains the most prevalent anthropometric measurement for obesity, it has limitations as it does not differentiate between lean and fat mass. WC has been introduced as an alternative for indirectly assessing the increase in visceral fat, serving as a marker for abdominal obesity and aiding in the diagnosis of metabolic syndrome (5). However, similar to BMI, WC on its own is incapable of differentiating between visceral and subcutaneous fat masses. In fact, the accuracy of WC in estimating abdominal fat mass is enhanced when BMI is included as an explanatory variable (6). Weight-adjusted waist index (WWI), which Park et al. (7), is an innovative measure of abdominal obesity that normalizes WC by body weight, leveraging the advantages of WC while reducing its correlation with BMI. The WWI serves not only to differentiate between adipose and muscular tissues but also to address issues of central obesity that are not directly related to overall body weight (8). As reported by Kim et al. (9), there was a positive correlation between both BMI and WC with measurements of fat and muscle. In contrast, the WWI showed a positive relationship with overall and abdominal fat measurements, while it exhibited a negative correlation with the mass of appendicular skeletal muscle (9). In a 40-month longitudinal study involving 1,946 participants, computed tomography (CT) data revealed a rise in measurements of abdominal fat along with a corresponding decline in muscle mass, concurrent with an escalation in the WWI (10). In addition, previous studies had identified a notable association between elevated WWI values and various health issues, including non-alcoholic fatty liver disease (11), diabetic kidney disease (12), bone mineral density (13), and depression (14).

Flavonoid, derived from the Latin term ‘flavus’ which signifies the color yellow, is omnipresent in the plant kingdom and represent the most prevalent polyphenolic substances in the human diet (15). A vast array of naturally occurring flavonoids, numbering over 5,000, have been identified in a variety of plant species. These compounds feature a 15-carbon (C6–C3–C6) backbone structure, with two benzene rings linked by a straight 3-carbon chain. The classification of flavonoid into multiple subcategories is based on the substitution patterns of the C ring, with further differentiation within each class achieved through the A and B substitutions (16). The main subgroups of flavonoid include flavonols, flavanones, isoflavones, flavones, flavans-3-ols, and anthocyanins. Over the past two decades, flavonoid compounds have been extensively studied for their potential positive effects on human health. Dietary flavonoid intake has been shown to be significantly and negatively associated with hepatic steatosis and fibrosis (17), metabolic syndrome (18), and obesity (19). The anti-obesity effects of flavonoid is believed to operate through a variety of mechanisms, such as inhibiting enzymes, modulating neuro-hormonal responses related to food consumption and satiety, and promoting the generation of mitochondria (20). So, can flavonoids compounds naturally prevent or reduce abdominal obesity? Currently, there is a lack of research regarding the potential of flavonoids consumption in reducing the risk of developing abdominal obesity. Hence, the importance of this study lies in investigating the effects of dietary flavonoids intake on WWI using data from the National Health and Nutrition Examination Survey (NHANES) database, and uncovering the potential of flavonoids compounds in reducing the risk of abdominal obesity.

## 2 Methods

### 2.1 Study population

**Figure 1:**
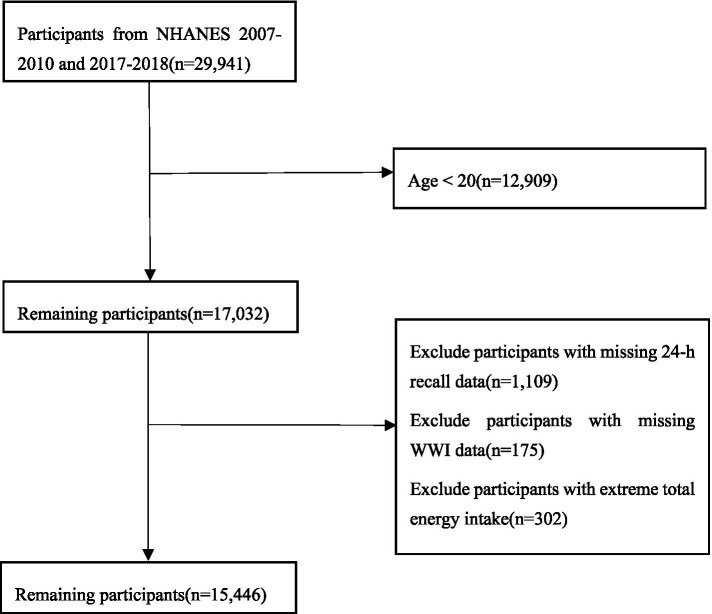
Sample inclusion flow.

NHANES, overseen by the National Center for Health Statistics (NCHS), is a comprehensive, nationwide survey aimed at assessing the health of the American population. The NCHS Ethics Review Board has endorsed the stratified multistage sampling approach employed in the survey. NHANES data collection involved an initial in-home interview, followed by a health examination at mobile examination center (MEC), and concludes with a follow-up telephone interview. For this study, we utilized data from the survey periods of 2007–2010 and 2017–2018. All participants were required to provide written consent after receiving comprehensive information about the study. Our study included 17,032 participants above the age of 20 and collected information on their flavonoids intake and WWI values. After careful consideration, participants with missing 24-h recall data (*n* = 1,109) were removed from the study. Participants who had missing WC and weight data (*n* = 175) were omitted, too. Furthermore, a total of 302 participants were excluded as they had either extremely low or high energy intakes: < 500 kcal or > 5,000 kcal/day for females and < 500 kcal or > 8,000 kcal/day for males. Finally, 15,446 participants were selected for inclusion (Figure 1).

### 2.2 Dietary assessment of flavonoids

The United States Department of Agriculture (USDA) Food Codes’ Flavonoid Database for the years 2007–2010 and 2017–2018 offers detailed information on the flavonoid content of a wide array of foods and beverages, as listed in the USDA and Food and Nutrient Database for Dietary Studies (FNDDS) version 5.0. The database contains detailed information on 29 specific flavonoids, which are divided into six categories: Anthocyanidins, Flavan-3-ols, Flavanones, Flavones, Flavonols, and Isoflavones, aligning with the dietary data provided by NHANES for the years 2007–2010 and 2017–2018. To determine the average daily flavonoids intake during these time-frames, a two-day dietary recall approach was utilized, involving in-person interviews with participants to document their food consumption over 2 days, supplemented by a subsequent telephone interview approximately 1 week later. In this study, flavonoids intake was determined solely from dietary sources, including water, without consideration for the consumption of flavonoid supplements or pharmaceuticals.

### 2.3 WWI

WWI (cm/√kg) is calculated by dividing WC (cm) by the square root of the individual’s weight (kg). These body measurements were conducted at MEC, with a health technologist responsible for recording and documenting the data. The current anthropometric standards were used to measure weight and WC (21). The participants were asked to wear specific attire for the assessment and then position themselves in the center of a digital scale, barefooted, with arms resting by their sides and their gaze directed straight ahead. WC was determined using a measuring tape, with MEC health technicians marking a horizontal line above the superior lateral edge of the right ilium. A right midaxillary line was then drawn, and the tape was positioned horizontally at the intersection of these two lines. Technicians, standing on the participant’s right side, wrapped the tape around the torso in a horizontal plane.

### 2.4 Covariates

The survey’s covariates encompassed demographic factors such as age, gender, ethnicity, educational attainment, and the family poverty income ratio (PIR), alongside lifestyle factors including alcohol and tobacco consumption, physical activity levels, and dietary intake of calories, carbohydrates, proteins, fats, and fiber. We categorized education levels as follows: below high school (below 11th grade), high school (graduated from high school or obtained a GED equivalent), and beyond high school (completed college or achieved a higher level of education). Poverty status was categorized into two groups based on income level: PIR <130% referred to low income, while PIR ≥130% designated high income (22). Smoking status was categorized into three groups: never smoked (consuming fewer than 100 cigarettes throughout their life), former smokers (having smoked over 100 cigarettes but are currently abstaining), and current smokers (who have smoked over 100 cigarettes and continue to smoke either occasionally or regularly). Alcohol consumption status was categorized into three groups: lifetime abstainer (who have consumed alcohol less than 12 times in their lifetime), former drinker (who have consumed alcohol more than 12 times in their lifetime but have not consumed it in the past year) and current drinker (who currently consume alcohol more than three times a week). Physical activity was categorized into two levels: low, defined as less than 599 metabolic equivalents (METs) per week, and high, defined as 599 METs or more per week (23).

### 2.5 Statistical analysis

Taking into account the complex sampling strategy of NHANES, we applied appropriate weights to the data in accordance with NHANES guidelines, which involved using a third of the weights from both the 2007–2010 and 2017–2018 periods. In this study, 15,446 individuals were adjusted for accuracy to represent a population of 218,690,615. Continuous data were presented as the mean with the standard deviation, whereas categorical data were represented as unweighted numbers (weighted percentages). The participant characteristics were analyzed according to tertile of total flavonoids intake, using weighted student’s t-tests for continuous variables and weighted chi-squared tests for categorical variables. The levels of flavonoids consumption were divided into three groups (T1, T2 and T3) and employed weighted generalized linear regression models with the low-intake group (T1) as the reference group. The crude model did not take into account any of the variables, whereas model 1 adjusted for potential confounding factors such as age, gender, ethnicity, education levels, and PIR. After considering physical activity levels, total carbohydrate intake, total protein intake, total fat intake, and total fiber intake, we made additional adjustments in model 2. We selected the above covariates based on professional knowledge, previously identified risk factors for WWI (7, 9, 10), and the observed incomparability of participant characteristics (Table 1). We decided to exclude the other covariates for the reasons that preliminary analyses indicated potential collinearity between them. Furthermore, to maintain model parsimony and avoid overfitting, we aimed to include only above covariates that had the most substantial and theoretically justified impact on the relationship between flavonoid intake and WWI. Restricted cubic splines (RCS) were utilized in Model 2 to explore the possible non-linear relationships between flavonoids intake and WWI. Furthermore, in order to examine the influence of total flavonoids intake on WWI, subgroup analyses and interaction exploration were performed to assess the effect of covariates that exhibited notable variations across different levels of total flavonoids intake. All subgroup variable analyses performed using a fully adjusted Model 2, and generalized linear regression models were additionally applied to assess the interactive effects between these subgroups and WWI. The missing covariate values were imputed by the “mice” package in R. The nationally representative estimates in NHANES were calculated by utilizing “SDMVPSU”and “SDMVSTRA.” The data processing was done in R version 4.3.1. A statistically significant difference was determined by considering a two-sided *p*-value <0.05.

**Table 1:** Basic characteristics of total participants stratified by total flavonoids tertile.

Characteristics	Total (*n* = 15,446)	Category of Flavonoid Intake, mg/100 g foods/day			*p*-value
		T1 (*n* = 5,380)	T2 (*n* = 5,384)	T3 (*n* = 4,682)	
Age (year)	47.35 (16.96)	45.02 (17.05)	48.14 (17.22)	48.88 (16,34)	<0.001
Gender, *n* (%)					0.3
Male	7,533 (47.83%)	2,623 (48.59%)	2,654 (48.31%)	2,257 (46.61%)	
Female	7,913 (52.17%)	2,757 (51.41%)	2,730 (51.69%)	2,425 (53.39%)	
Ethnicity, *n* (%)					<0.001
Mexican-American	2,551 (8.61%)	979 (10.18%)	1,035 (10.30%)	537 (5.34%)	
Non-Hispanic White	6,847 (67.03%)	2,309 (65.75%)	2,203 (64.14%)	2,334 (71.19%)	
Non-Hispanic Black	3,149 (11.22%)	1,222 (12.99%)	1,050 (11.30%)	877 (9.38%)	
Others	2,901 (13.14%)	870 (11.08%)	1,096 (14.26%)	934 (14.09%)	
Poverty status, *n* (%)					<0.001
No	9,658 (72.42%)	3,092 (68.28%)	3,383 (73.37%)	3,181 (75.61%)	
Yes	5,790 (27.58%)	2,288 (31.72%)	2,001 (26.63%)	1,501 (24.39%)	
Education levels, *n* (%)					<0.001
More than high school	7,726 (58.49%)	2,184 (48.93%)	2,831 (62.54%)	2,709 (63.98%)	
High school	3,684 (25.43%)	1,458 (30.70%)	1,184 (21.80%)	1,042 (23.81%)	
Less than high school	4,038 (16.08%)	1,738 (20.37%)	1,369 (15.66%)	931 (12.21%)	
Smoking status, n (%)					<0.001
Never	8,436 (55.21%)	2,597 (48.34%)	3,128 (59.04%)	2,709 (58.24%)	
Former	3,829 (24.65%)	1,311 (23.72%)	1,360 (25.35%)	1,158 (24.89%)	
Current	3,180 (20.14%)	1,472 (27.94%)	895 (15.61%)	813 (16.87%)	
Drinking status, *n* (%)					0.006
Never	3,241 (16.23%)	1,087 (15.29%)	1,098 (16.29%)	1,055 (17.12%)	
Former	3,062 (16.84%)	1,190 (19.20%)	1,009 (15.30%)	862 (16.01%)	
Current	9,146 (66.93%)	3,103 (65.51%)	3,277 (68.41%)	2,765 (66.87%)	
Physical activity, *n* (%)					0.001
High	9,137 (65.02%)	2,963(62.51%)	3,275 (65.66%)	2,898 (66.88%)	
Low	6,311 (34.98%)	2,417 (37.49%)	2,109 (34.34%)	1,786 (33.12%)	
BMI, *n* (%)					<0.001
BMI < 25	4,234 (29.18%)	1,368 (26.33%)	1,497 (29.48%)	1,369 (31.75%)	
BMI ≥ 25	1,1214 (70.82%)	4,012 (73.67%)	3,887 (70.52%)	3,313 (68.25%)	
Weight (kg)	82.86 (21.83)	84.28 (22.44)	82.46 (21.62)	81.83 (21.34)	0.003
WC (cm)	99.13 (16.67)	100.31 (16.93)	98.71 (16.58)	98.37 (16.44)	<0.001
WWI (cm/√kg)	10.91 (0.90)	10.95 (0.90)	10.90 (0.89)	10.89 (0.90)	0.034
Total calories (kcal/d)	2,095.41 (837.56)	1,930.17 (785.30)	2,183.91 (870.05)	2,172.47(830.56)	<0.001
Total carbohydrate (g)	249.87 (108.69)	225.19 (102.75)	259.14 (106.27)	265.31 (112.51)	<0.001
Total protein (g)	81.83 (35.18)	75.82 (33.20)	85.25 (36.73)	84.43 (34.75)	<0.001
Total fat (g)	81.26 (39.58)	77.42 (38.63)	83.14 (41.43)	83.24 (38.32)	<0.001
Total fiber (g)	16.76 (9.03)	12.96 (6.46)	18.61 (8.76)	18.71 (10.21)	<0.001
Total flavonoids (mg)	227.90 (410.82)	17.50 (9.47)	78.79 (33.90)	587.41 (556.19)	<0.001
Flavonols (mg)	18.96 (18.73)	7.83 (5.30)	15.38 (10.11)	33.67 (23.86)	<0.001
Flavanones (mg)	12.60 (26.63)	1.35 (3.80)	18.94 (24.76)	17.53 (36.19)	<0.001
Isoflavones (mg)	2.14 (11.40)	0.54 (2.08)	2.65 (10.20)	3.24 (16.67)	<0.001
Flavones (mg)	0.95 (2.10)	0.49 (0.74)	1.00 (1.44)	1.35 (3.19)	<0.001
Flavan-3-ols (mg)	178.95 (391.91)	5.37 (5.21)	25.32 (27.68)	506.17 (547.01)	<0.001
Anthocyanidins (mg)	14.30 (37.50)	1.92 (3.74)	15.51 (20.84)	25.46 (59.10)	<0.001

T, Tertile; BMI, body mass index; WC, weight circumference; WWI, Weight-adjusted waist index; continuous data were displayed as weighted means (standard deviation) and categorical variables were exhibited as unweighted numbers (weighted percentages).

## 3 Results

### 3.1 Characteristics of participants

The study involved 15,446 participants with an average age of 47.35 ± 16.96 years, of whom 7,533 (47.83%) were male and 7,913 (52.17%) were female. The average WWI across all participants was 10.91 ± 0.90 cm/√kg. The mean total flavonoids intake for all participants was 227.90 ± 410.82 mg/100 g foods/day, with the values for the different tertiles as follows: T1: 0–36.08 mg; T2: 36.08–160.52 mg; T3: 160.52–8,018.67 mg. Participants in the highest tertile of total flavonoids intake were older, Non-Hispanic White, had a higher level of education, higher PIR, smoked less, and consumed alcohol more frequently. Additionally, higher intakes of total flavonoids were more likely to with higher physical activity, total caloric intake, carbohydrate, protein, fat consumption, as well as fiber intake (Table 1).

### 3.2 Associations between dietary flavonoids intake and WWI

**Figure 2:**
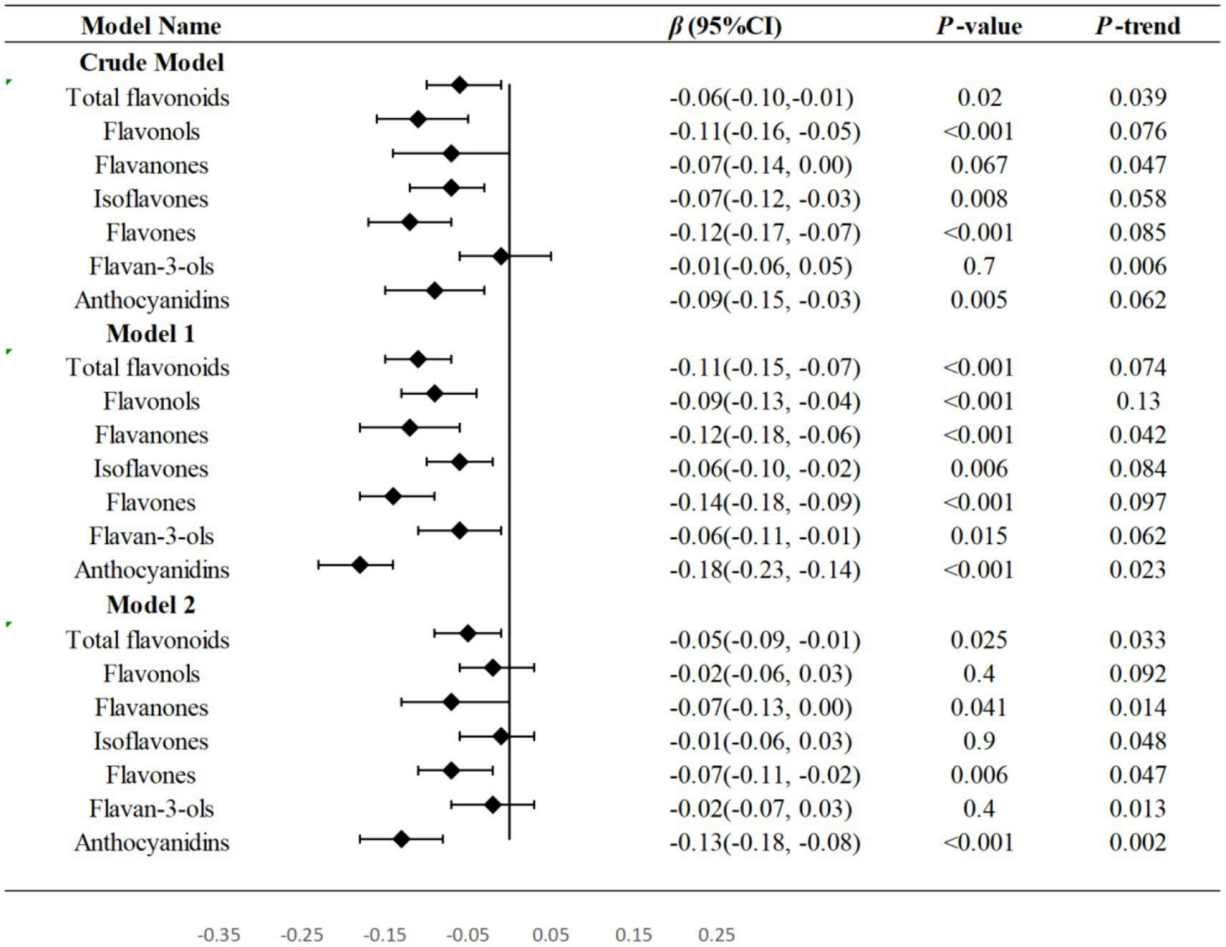
Forest plots were used to show the relationship between the third tertile of flavonoids and WWI in three models and linear trend test was conducted by treating each flavonoid as a continuous variable. T Tertile; WWI Weight-adjusted waist index; PIR family income to poverty. Model 1: no covariates were adjusted. Model 2: adjusted for age, gender, ethnicity, education levels and PIR. Model 3: adjusted for age, gender, ethnicity, education levels, PIR, physical activity levels, total carbohydrate intake, total protein intake, total fat intake and total fiber intake.

Table 2 displayed the outcomes of weighted linear regression analyses that evaluate the relationships between total flavonoids and flavonoid subclass intake with WWI. In model 2, A statistically significant negative correlations were observed between total flavonoids, flavanones, flavones, flavan-3-ols, and anthocyanidins (*p* = 0.025, *p* = 0.041, *p* = 0.006, *p* = 0.0.035, *p* < 0.001, respectively) with WWI after adjusting demographic variables and lifestyle variables except flavonols and isoflavones. Notably, when using the first tertile as the reference, the third tertile of total flavonoids (*β*: -0.05, 95%CI: −0.09, −0.01), flavanones (*β*: -0.07, 95%CI: −0.13, 0.00), flavones (*β*: -0.07, 95%CI: −0.11, −0.02), anthocyanidins (*β*: -0.13, 95%CI: −0.18, −0.08), and the second tertile of flavan-3-ols (*β*: -0.06, 95%CI: −0.11, 0.00) had demonstrated the most significant potential for reducing WWI. In all three models, there was a significant negative correlation seen between T3 of total flavonoids (all *p* < 0.05), T2 and T3 of flavones (*p* < 0.01), T2 of flavan-3-ols (*p* < 0.05), T3 of anthocyanidins (*p* < 0.01) and WWI. Forest plot was used to show the relationship between the third tertile of flavonoids and linear trend test was conducted by treating each flavonoid as a continuous variable. In model 2, total flavonoids, flavanones, flavones, and anthocyanidins indicted a linear trend with WWI (*P*-trend = 0.033, *P*-trend = 0.014, *P*-trend = 0.047, *P-*trend = 0.002, respectively) (Figure 2).

**Table 2:** Associations between flavonoids intake and WWI.

	Crude Model β (95% CI)	*p*-value	Model 1 β (95% CI)	*p*-value	Model 2 β (95% CI)	*p*-value
Total flavonoids(mg)						
T1(0–36.08 mg)	Reference	–	Reference	–	Reference	–
T2 (> 36.08, ≤ 160.52)	0.05 (−0.10, 0.00)	0.064	−0.10 (−0.14, −0.06)	<0.001	−0.04 (−0.08, 0.00)	0.040
T3 (> 160.52, ≤ 8,018.67)	−0.06 (−0.10, −0.01)	0.020	−0.11 (−0.15, −0.07)	<0.001	−0.05 (−0.09, −0.01)	0.025
Flavonols (mg)						
T1 (0–9.31)	Reference	–	Reference	–	Reference	–
T2 (> 9.31, ≤ 20.01)	−0.03 (−0.08, 0.02)	0.300	−0.03 (−0.07, 0.02)	0.200	0.01 (−0.03, 0.06)	0.500
T3 (> 20.01, ≤ 332.04)	−0.11 (−0.16, −0.05)	<0.001	−0.09 (−0.13, −0.04)	<0.001	−0.02 (−0.06, 0.03)	0.400
Flavanones (mg)						
T1 (0–0.14)	Reference	–	Reference	–	Reference	–
T2 (> 0.14, ≤ 4.17)	−0.07 (−0.14, 0.00)	0.053	−0.09 (−0.15, −0.02)	0.009	−0.06 (−0.12, 0.00)	0.054
T3 (> 4.17, ≤ 590.62)	−0.07 (−0.14, 0.00)	0.067	−0.12 (−0.18, −0.06)	<0.001	−0.07 (−0.13, 0.00)	0.041
Isoflavones (mg)						
T1 (= 0)	Reference	–	Reference	–	Reference	–
T2 (> 0, ≤ 0.01)	0.01 (−0.06, 0.07)	0.031	−0.03 (−0.08, 0.02)	0.200	−0.02 (−0.07, 0.03)	0.700
T3 (> 0.01, ≤ 390.60)	−0.07 (−0.12, −0.03)	0.008	−0.06 (−0.10, −0.02)	0.006	−0.01 (−0.06, 0.03)	0.900
Flavones (mg)						
T1 (0–0.28)	Reference	-	Reference	-	Reference	-
T2 (> 0.28, ≤ 0.90)	−0.09 (−0.13, −0.04)	<0.001	−0.09 (−0.13, −0.06)	<0.001	−0.06 (−0.10, −0.03)	0.002
T3 (> 0.90, ≤ 87.25)	−0.12 (−0.17, −0.07)	<0.001	−0.14 (−0.18, −0.09)	<0.001	−0.07 (−0.11, −0.02)	0.006
Flavan-3-ols (mg)						
T1 (0–8.19)	Reference	–	Reference	–	Reference	–
T2 (> 8.19, ≤ 66.11)	−0.08 (−0.14, −0.01)	0.018	−0.11 (−0.16, −0.05)	<0.001	−0.06 (−0.11, 0.00)	0.035
T3(> 66.11, ≤ 7,727.37)	−0.01 (−0.06, 0.05)	0.700	−0.06 (−0.11, −0.01)	0.015	−0.02 (−0.07, 0.03)	0.400
Anthocyanidins (mg)						
T1 (0–0.405)	Reference	–	Reference	–	Reference	–
T2 (> 0.405, ≤ 6.92)	−0.01 (−0.06, 0.05)	0.700	−0.08 (−0.13, −0.04)	<0.001	−0.06 (−0.11, −0.02)	0.011
T3 (> 6.92, ≤ 1,497.9)	−0.09 (−0.15, −0.03)	0.005	−0.18 (−0.23, −0.14)	<0.001	−0.13 (−0.18, −0.08)	<0.001

T, Tertile; WWI, Weight-adjusted waist index; PIR, family income to poverty. Weighted generalized linear regression models for the association between flavonoids intake and WWI. Model 1: no covariates were adjusted. Model 2: adjusted for age, gender, ethnicity, education levels and PIR. Model 3: adjusted for age, gender, ethnicity, education levels, PIR, physical activity levels, total carbohydrate intake, total protein intake, total fat intake and total fiber intake.

### 3.3 The non-linear relationships between flavonoids intake and WWI

**Figure 3:**
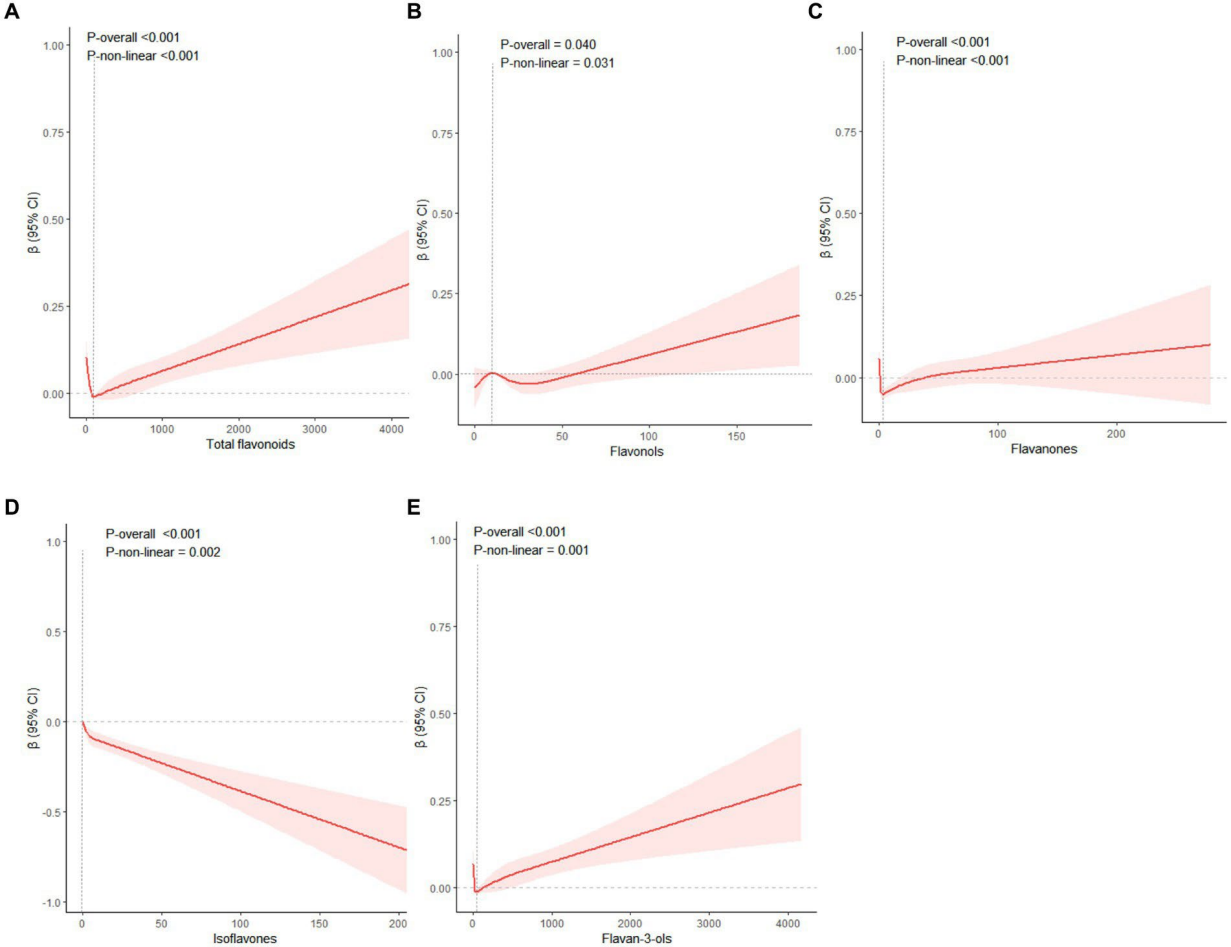
The restricted cubic splines were used to display the association between total flavonoids **(A)**, flavonols **(B)**, flavanones **(C)**, isoflavones **(D)**, flavan-3-ols (E) with WWI. Adjusted for age, gender, ethnicity, education levels, PIR, physical activity levels, total carbohydrate intake, total protein intake, total fat intake and total fiber intake. WWI Weight-adjusted Waist index; PIR family poverty income ratio.

We utilized an RCS to examine the non-linear relationships between the intake of flavonoids and WWI, with the median consumption of all flavonoids serving as the baseline for comparison. In Model 2, it was determined that total flavonoids (*P* for non-linearity < 0.001), flavonols (*P* for non-linearity = 0.031), flavanones (*P* for non-linearity < 0.001), isoflavones (*P* for non-linearity = 0.002), and flavan-3-ols (*P* for non-linearity = 0.001) exhibited a statistically significant non-linear association with WWI. Association between total flavonoids, flavonols, flavanones, flavan-3-ols and WWI was J-shaped curve. The WWI showed a consistent decline with the increment in total flavonoids intake, up to a point where the intake reached 108.86 mg/100 g foods/day and the consumption rage of total flavonoids in 65.43–217.45 mg/100 g foods/day have the ability to decrease WWI. Beyond this rage, WWI started to rise with further increases in total flavonoids consumption. Within the dietary intake range of <8.39 mg/100 g foods/day and 12.12–57.81 mg/100 g foods/day, flavonols exhibited a capacity to decrease WWI and the optimal reduction occurring at a level of 30.77 mg/100 g foods/day. At 2.80 mg/100 g foods/day (flavanones) and 41.85 mg/100 g foods/day (flavan-3-ols) points, WWI values were the lowest, respectively. Additionally, beyond the rage of flavanones (1.40–40.63 mg/100 g foods/day) and flavan-3-ols (20.92–104.64 mg/100 g foods/day), WWI started to increase. On the contrary, when isoflavones intake increased, WWI values consistently dropped, and the downward trend then slightly increased (Figure 3).

### 3.4 Subgroup and interaction analysis

**Figure 4:**
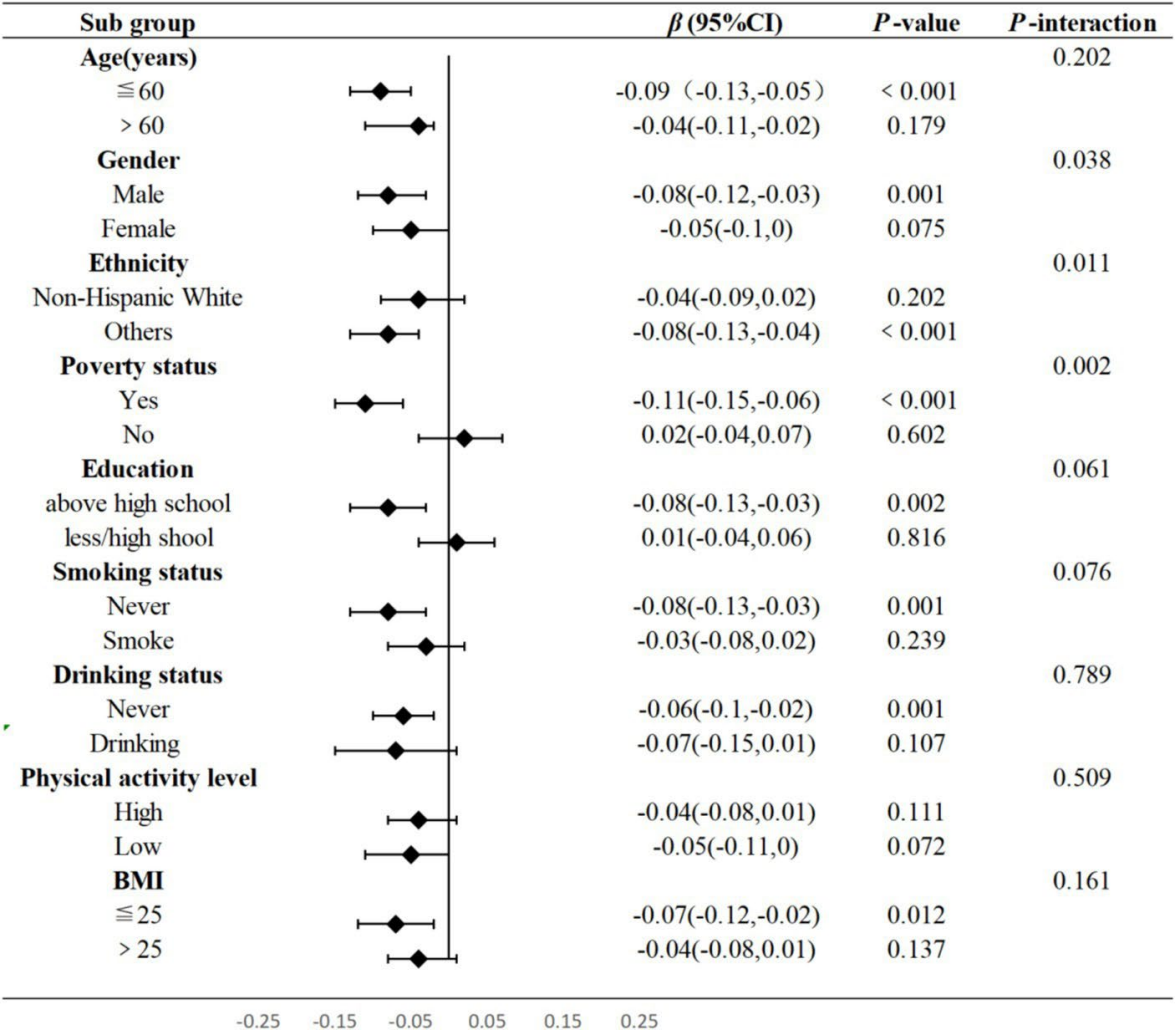
Subgroup and interaction analysis between total flavonoids and WWI. Adjusted for age, gender, ethnicity, education levels, PIR, physical activity levels, total carbohydrate intake, total protein intake, total fat intake and total fiber intake. T, Tertile, BMI, Body Mass Index, WWI, Weight-adjusted Waist, PIR, family poverty income ratio.

Figure 4 presented the subgroup and interaction analysis between WWI and total flavonoids intake, revealing significant interactions with respect to races, gender, and poverty status (*P* for interaction = 0.011, P for interaction = 0.038, P for interaction = 0.002, respectively). When compared to Non-Hispanic White, the intake of total dietary flavonoids appeared to have a more pronounced protective effect against obesity in other populations (*p* < 0.001). Notably, a higher intake of total flavonoids was linked to a lower WWI among males (*p =* 0.001) and individuals in poverty (*p* < 0.001).

## 4 Discussion

Based on a representative sample of 15,446 US adults, the present analyses showed that the average consumption of total flavonoids was 227.90 mg/100 g foods/day mostly from flavan-3-ols (178.95 mg/100 g foods/day) and flavonols (18.96 mg/100 g foods/day). Our findings revealed that WWI decreased with increasing levels of dietary intake of total flavonoids, flavanones, flavones, flavan-3-ols, and anthocyanidins after adjusting demographic variables and lifestyle variables. Additionally, the RCS curves demonstrated that the intake of total flavonoids, flavonols, flavanones, and flavan-3-ols within a certain range can exert the greatest effect in reducing WWI levels. Subgroup and interaction analysis further revealed that other ethnic groups (Mexican American and Others), male, and participants living in poverty had a stronger negative correlation between the total amount of flavonoids and WWI.

Flavonoids, a class of polyphenolic compounds unique to the plant kingdom, have recently sparked significant interest due to their potential health benefits for humans. Notable sources of these dietary components encompass a variety of plant-based foods, such as tea, berries, citrus fruits, and soy products (24). In the present study, tea emerged as the predominant source for the total flavonoids, flavonols, and flavan-3-ols categories. Meanwhile, a diverse array of foods significantly contributed to the intake of other flavonoid classes, with orange juice being a key source of flavanones, protein powders for isoflavones, sweet peppers for flavones and blueberries for anthocyanins (25). Kim et al. (26) examined the relationship between the regular consumption of various flavonoids categories and obesity in 23,118 adults in Korea found that a higher total intake of flavonoid was associated with a lower prevalence of obesity in women (OR = 0.82; 95%CI: 0.71–0.94) and the consumption of flavonols (OR = 0.88; 95%CI:0.78–0.99), isoflavones (OR = 0.85; 95%CI:0.75–0.96) and proanthocyanidins (OR = 0.81; 95%CI:0.71–0.92) was found to be inversely associated with the risk of abdominal obesity. However, the intake of flavonols (OR = 1.16; 95%CI:1.02–1.33), flavanones (OR = 1.18; 95%CI:1.04–1.35), and anthocyanidins (OR = 1.27; 95%CI:1.11–1.46) was positively correlated with obesity as determined by BMI in males (26). In our research, we found that flavanones (β:-0.07; 95%CI: −0.13, 0.00) and flavones (β:-0.07; 95%CI:-0.11, −0.02) were more effective in reducing WWI and male participants had a stronger negative correlation between the total amount of flavonoids and WWI. This disparity could be attributed to the fact that the authors of the referenced study utilized the Korean health and nutrition survey, which may have a predisposition towards abdominal obesity and distinct dietary pattern (kimchi was the main source of flavonoid intake) (27, 28). What is more, abdominal obesity was defined by WC in the research of Kim et al. (28), while in the presented study, the WWI was to describe abdominal obesity. Quercetin is the most commonly found individual flavonols in daily dietary intake. The research conducted by Nishimura et al. (29) indicated that quercetin intake was inversely correlated with fat mass, BMI and WC in central obese participants. However, daily quercetin supplement of 60 mg did not alter the overall abdominal fat content in a healthy population (29). Additional research has indicated that the administration of high doses of quercetin (500 or 1,000 mg) did not influence body mass or composition among healthy individual (30). These findings were different from the outcomes reported in our study that the dietary flavonols within a certain rage (12.12–57.81 mg/100 g foods/day) had the ability to attenuated WWI. This could imply that the effects of quercetin on certain parameters might be predominantly observed in individuals with higher body mass or when combined with other flavonols, as these also showed potential effects on obesity-related measures.

In our subgroup analysis, we identified that certain demographic groups may experience greater benefits from the intake of total flavonoids. Given the multi-ethnic composition of the United States, our study revealed that total flavonoids consumption has a particularly significant impact on populations other than Non-Hispanic Whites, notably among Mexican-Americans (*p* = 0.007) and individuals of Others (*p* < 0.001) ethnic backgrounds. This could be attributed to variations in lifestyle, dietary practices, and genetic predispositions across different racial and ethnic groups (31). It is possible that Non-Hispanic Whites have a reduced sensitivity to the effects of flavonoids, although additional research is necessary to confirm this hypothesis. With an increase in total flavonoids intake, we observed a trend of decreasing WWI among male participants. The potential mechanisms behind these associations might be related to sex differences in body composition influenced by dietary consumption. Body fat distribution varies between genders, with men tending to accumulate a greater amount of fat in the abdominal region compared to women. This makes men more prone to developing abdominal obesity (32). Given the consistent rise in the incidence of abdominal obesity among men in the US (33), the inverse relationship between total flavonoids consumption and abdominal obesity in this demographic suggested that dietary recommendations emphasizing flavonoid-rich foods might be less effective in preventing abdominal obesity in women. Furthermore, a stronger correlation between total flavonoids intake and the WWI had been noted in individuals with PIR below 1.3. This could be attributed to disparities in the primary sources of flavonoids between lower and higher socioeconomic groups, with lower-income families potentially engaging more frequently in home-cooked meals, which may influence their dietary flavonoid intake (34, 35).

It is known that the pathogenesis of abdominal obesity was different from generalized obesity (36). Human omental preadipocytes cultured preadipocytes undergo lower adipogenic transcription factor expression and more TNF-a-induced apoptosis than human abdominal subcutaneous. Additionally, there are preadipocytes within visceral fat that have limited ability to proliferate and differentiate compared to subcutaneous fat (37). Omental adipocytes have been observed to display comparable or reduced levels of basal lipolysis and exhibit decreased sensitivity to the insulin-induced antilipolytic effects *in vivo* (38). In fact, there is a higher level of glucose absorption in visceral fat in comparison to subcutaneous fat (39). Furthermore, the omental fat demonstrates a higher capacity to absorb plasma free fatty acids compared to the abdominal subcutaneous fat, which suggests that this specific process might play a role in the selective buildup of visceral fat (40). For our research, we observed a strong inverse relationship between the consumption of total flavonoids, flavanones, flavones, flavan-3-ols, and anthocyanidins with WWI. This finding indicated that a sufficient intake of above flavonoids could potentially be a mildly effective intervention for combating abdominal obesity. Naringenin (4′,5,7-thrihydroxyflavanone) is a flavanone commonly found in a variety of vegetables, fruits, herbs, and nuts that are widely consumed by humans (41, 42). Research conducted by Atsuyoshi et al. had shed light on the anti-diabetic properties of naringenin. Their findings indicated that naringenin can increase the secretion of adiponectin and reduce the size of 3 T3-L1 cells in a concentration-dependent manner during the process of adipocyte differentiation (43). Adiponectin is significant for its role in enhancing glucose uptake without the need for insulin receptor stimulation, improving fatty acid metabolism, and reducing fatty acid levels, which in turn increases the sensitivity of the insulin receptor. Additionally, it promotes insulin sensitivity through the activation of liver AMP-activated protein kinase (AMPK), prevents arteriosclerosis, exhibits anti-inflammatory effects, and suppresses myocardial hypertrophy (44). Baicalein, which root constitute flavones baicalein (45), has been demonstrated in multiple studies to effectively alleviate insulin resistance and ameliorate complications associated with obesity (46, 47). Additionally, baicalein had been shown to exert protective effects on the functionality of INS382/13 cells and human pancreatic islets, thereby enhancing their secretory capacity (48). Luteolin, a widely available flavone in the diet, had also been identified for its potential anti-obesity characteristics (49). According to research by Lin et al. (50), luteolin was capable of diminishing fat storage in *Caenorhabditis elegans* by stimulating central serotonin signaling, which triggers fat loss. Treatment with luteolin led to an increase in serotonin synthesis within ADF neurons and the subsequent activation of serotonin-linked receptors MOD-1 and SER-6. This activation sequence ultimately results in an upregulation of lipolysis and fatty acid β-oxidation in the nematode model, *Caenorhabditis elegans* (50). Flavan-3-ols, encompassing the catechin group of compounds, are characterized by a 2-phenyl-3,4-dihydro-2H-chromen-3-ol framework. Green tea is particularly abundant in catechins, including epigallocatechin 3-gallate (EGCG) and catechin 3-gallate, which are both classified under flavan-3-ols (51). The impact of catechins and EGCG on abdominal obesity and metabolic syndrome has been a focus of extensive research, particularly in animal models. For instance, a study involving mice on a high-fat diet, which constituted 60% of their caloric intake, demonstrated that a dietary regimen incorporating EGCG at a concentration of 0.32% for 16 weeks led to significant reductions in body weight gain, overall body fat, and visceral fat weight when compared to the control group without EGCG supplementation (52). Zang et al. (53) conducted research on the effects of green tea extract in juvenile and adult zebrafish models of obesity. Their findings indicated that green tea extract not only significantly reduced the accumulation of visceral adipose tissue in juveniles but also improved visceral adiposity and plasma triglyceride levels in adult zebrafish. The proposed molecular mechanism behind the anti-obesity effects of green tea extract involves the activation of the Wnt/β-catenin and AMPK signaling pathways, which may help to ameliorate obesity-related phenotypes (53). Multiple studies indicated that the transition of preadipocytes into adipocytes is governed by a tightly regulated sequence of transcriptional events, with proliferator-activated receptor gamma (PPARγ) and CCAT/enhancer-binding proteinα (C/EBPα) being pivotal mediators in this process (54, 55). PPARγ functions as a regulator in the lipid synthesis pathway, and its overexpression leads to an enlargement and multiplication of lipid molecules within 3 T3-L1 preadipocytes undergoing differentiation (56). The black soybean contains various anthocyanins like cyanidin-3-O-glucoside, and delphinidin-3-O-glucoside, which have significant implications. They have been studied to increase preadipocyte differentiation through downregulated the expression of PPARγ and C/EBPα in a concentration-dependent manner (57). Additionally, anthocyanins can stimulate lipolysis in mature adipocytes. Oral administration of cyanidin had been shown to decrease hepatic and plasma triglyceride levels, reduce adiposity, and enhance glucose tolerance in mice on a high-fat diet. Metabolomic analysis of the liver indicated that cyanidin influenced the metabolic profile towards increased fatty acid oxidation and ketogenesis (58).

The robustness and broad applicability of our research findings were bolstered by the large and diverse sample size, enabling us to detect subtle association between dietary flavonoids consumption and abdominal obesity within multiple subgroups. Our study suggested that dietary flavonoids could pave the way for creating functional foods to combat abdominal obesity. However, it is crucial to recognize and address the limitations inherent in our study. Initially, our research was confined to examining the link between flavonoids consumption and the WWI, and further investigation is required to confirm the correlation between flavonoid intake and other metabolic health markers. Additionally, different dietary sources (foods/drinks) affect flavonoids bioavailability, the intake data may overestimated the actual amount of flavonoids intake that exhibit physiological health benefits. Lastly, the exclusion of supplement data may limit the generalizability of our findings, as it does not account for all possible sources of flavonoids that participants may have consumed. Future studies should consider including data on the use of flavonoids supplements to provide a more comprehensive evaluation of total flavonoids intake.

## 5 Conclusion

Our research contributed novel insights into the intricate relationship between flavonoids consumption and abdominal obesity, with our findings indicating a significant inverse correlation between flavonoids intake and the WWI among adults in the US. This underscored the potential significance of flavonoids intake in the management of abdominal obesity and laid a foundation for the development of dietary guidelines for flavonoid consumption. Nonetheless, the observed associations between flavonoids and WWI should not be interpreted as indicative of a causal relationship, and additional prospective cohort studies or randomized controlled trials are necessary to confirm the causal relationship between flavonoids intake and abdominal obesity, as well as to determine the safe and effective dosages of flavonoids for various populations.

## Data availability statement

The raw data supporting the conclusions of this article will be made available by the authors, without undue reservation.

## Ethics statement

The studies involving humans were approved by The National Centre for Health Statistics’ research ethics review board. The studies were conducted in accordance with the local legislation and institutional requirements. The participants provided their written informed consent to participate in this study.

## Author contributions

SZ: Writing – original draft. MY: Writing – original draft. XiL: Writing – original draft. HW: Writing – original draft. XuL: Writing – original draft. YF: Writing – original draft. DW: Funding acquisition, Writing – review & editing. BZ: Writing – review & editing.
