# Correction: Flavonoids intake and weight-adjusted waist index: insights from a cross-sectional study of NHANES

**DOI:** 10.3389/fnut.2026.1837976

**Published:** 2026-04-20

**Authors:** Shuang Zu, Meiling Yang, Xiude Li, Hanhan Wu, Xunliang Li, Yunshan Fan, Deguang Wang, Bao Zhang

**Affiliations:** 1 Department of Nephrology, The Second Affiliated Hospital of Anhui Medical University, Hefei, Anhui, China; 2 Department of Clinical Nutrition, The First Affiliated Hospital of Anhui Medical University, Hefei, Anhui, China; 3 Department of Gastroenterology, The First Affiliated Hospital of Anhui Medical University, Hefei, Anhui, China

**Keywords:** flavonoids intake, weight-adjusted waist index, NHANES, abdominal obesity, flavonoids dietary recommendation

In the published article, affiliation 1 “Department of Nephrology, The Second Affiliated Hospital of Anhui Medical University, Hefei, Anhui, China” was not correctly assigned to author Shuang Zu. Additionally, the order of affiliations was incorrect in the published article and have been revised accordingly. The correct affiliation list is as follows:

Shuang Zu^1, 2^, Meiling Yang^3^, Xiude Li^2^, Hanhan Wu^2^, Xunliang Li^1^, Yunshan Fan^2^, Deguang Wang^1*^, Bao Zhang^2*^

1. Department of Nephrology, The Second Affiliated Hospital of Anhui Medical University, Hefei, Anhui, China

2. Department of Clinical Nutrition, The First Affiliated Hospital of Anhui Medical University, Hefei, Anhui, China

3. Department of Gastroenterology, The First Affiliated Hospital of Anhui Medical University, Hefei, Anhui, China

In the published article, the wrong email address was assigned to corresponding author Bao Zhang. The correct email address is 596255538@qq.com.

The original version of this article has been updated.

